# Health technology assessment implementation in WHO South-East Asia Region: a realist review protocol

**DOI:** 10.12688/wellcomeopenres.19619.2

**Published:** 2024-07-02

**Authors:** Elstin Anbu Raj, Pragati Hebbar, Prashanth N Srinivas, Neethi V Rao, Nachiket Gudi, Angela Brand, Divya Sussana Patil

**Affiliations:** 1 Department of Health Information, Prasanna School of Public Health, Manipal Academy of Higher Education, Manipal, Karnataka, 576104, India; 2 Department of Pharmacy Practice, Manipal College of Pharmaceutical Sciences, Manipal Academy of Higher Education, Manipal, Karnataka, 576104, India; 3 Cluster on Chronic Conditions and Public Policies, Institute of Public Health Bengaluru, Bengaluru, Karnataka, 560070, India; 4 Health equity cluster, Institute of Public Health Bengaluru, Bengaluru, Karnataka, 560070, India; 5 Institute of Public Health Bengaluru, Bengaluru, Karnataka, 560070, India; 6 Independent Consultant, Bengaluru, India; 7 Faculty of Health Medicine and Life Sciences, Maastricht University, Maastricht, Limburg, 6200 MD, The Netherlands; 8 United Nations University – Maastricht Economic and Social Research Institute on Innovation and Technology, Maastricht University, Maastricht, Limburg, NL - 6211 AX, The Netherlands

**Keywords:** Health technology assessment, realist review, health technology interventions, evaluations, WHO-SEARO

## Abstract

**Background:**

A robust Health Technology Assessment (HTA) framework is crucial to address the rising burden of healthcare costs and to inform decision-making to promote high-quality health systems. This research aims to describe the HTA methods and mechanisms for the successful implementation of HTA in the WHO South-East Asia region, and contextualize the synthesized evidence relevant to Indian settings.

**Methods:**

Realist review involves developing a program theory by conducting a systematic search strategy, screening, study selection, data extraction, and data synthesis. A systematic search for literature will be conducted on PubMed (NCBI), EMBASE (Elsevier), Scopus (Elsevier), Web of Science (Clarivate), and ProQuest Central for identifying the methods used for HTA of health technology interventions. Stakeholder consultations will be conducted to develop a program theory following the Context-Mechanism-Outcome configurations (CMOcs) framework. Searches for primary evidence will be conducted iteratively. Data will be extracted and tested against the programme theory. The proposed realist review will be reported as per the Realist and MEta-narrative Evidence Syntheses: Evolving Standards [RAMESES II] guidelines.

**Conclusions:**

To our knowledge, there has been no comprehensive review conducted to understand the mechanisms of HTA methods in the WHO South-East Asia region. The findings from the realist review will help us understand the mechanisms through which the HTA could work in WHO South-East Asian countries. We will then contextualize the findings obtained from evidence to Indian settings, based on program theory development through stakeholder consultation. A framework will be developed that can be used by policymakers/HTA experts in India for effective implementation of the same.

## Introduction

Healthcare costs are not only ambiguous and arbitrary but can be disastrous to families living on the margins. The poor and vulnerable are not only forced to spend money out of pocket (OOP) due to ill health but also suffer from loss of wages to obtain medical care in Indian settings ( Downey *et al*., 2017). Global evidence shows that tax-based financing is the most progressive form of financing healthcare services, followed by social health insurance ( Gottret & Schieber, 2006; Xu *et al*., 2003). Since, healthcare is riddled with market failures, a purely market driven approach is inadequate to determine what to invest, for whom, and what outcomes are to be prioritized ( Elshaug *et al*., 2017). Given such a scenario, it is desirable to move towards a Universal Health Coverage (UHC) based health system where the complex and dynamic private sector is efficiently regulated, and market competition and choices are used as tools to enhance the quality of care and reduce the cost of care in Inida ( Chauhan & Agrawal, 2014).

There is a promising future for the healthcare system in India if an established and legitimate Health Technology Assessment (HTA) mechanism is in place to address the rising burden in healthcare costs and OOP expenditures of patients ( Dang *et al*., 2016). HTA is an important policy advice approach in health system for addressing inequities and inefficiencies by ensuring that resources are allocated efficiently and effectively ( MacQuilkan *et al*., 2018). Additionally, HTA serves as a bargaining tool between patients and providers to ensure healthcare decisions are more transparent, defensible, and affordable ( Kanavos *et al*., 2010). A survey conducted by World Health Organization (WHO) in 2015 found that approximately 80% of responding countries had a formal process for collecting evidence on new health technologies and services in their country ( World Health Organization, 2015). The need for HTA processes in Low- and Middle-Income countries (LMICs) will likely rise following the adoption of resolutions proposed during 2012-2014 by the WHO regional committee for South-East Asia ( World Health Organization, 2013).

A systematic economic evaluation can be valuable in helping LMICs achieve and maintain UHC. The use of HTA, normative choices, and methodological guidelines can differ among countries ( Culyer & Chalkidou, 2019). A leading example of harmonising the HTA landscape is the International Network of Agencies for Health Technology Assessment (INAHTA) ( Schwarzer & Siebert, 2009). However, determining health priorities requires consideration of the local context. WHO South-East Asia Regional Office (SEARO) comprises primarily of LMICs, whose health systems are diverse but lack research and evidence to guide decision making. ( Ghaffar *et al*., 2008). A large majority of LMICs suffer from inequalities in healthcare services as well as challenges related to the affordability and availability of basic healthcare services ( Peters *et al*., 2008). In addition, health policy and systems research are not recognized for their potential contribution to policy development ( Fraussen & Halpin, 2017). Furthermore, limited funding impedes the development of policy which is the need of the hour ( Majumder, 2004). Limited information on dedicated funding can also make the allocation of resources complicated and further stifles the growth of research in this area ( Majumder, 2004).

Developing frameworks, metrics, and methods is critical to knowledge creation and evidence-informed decision making. Research is often driven by medical institutions, universities, and civil societies in accordance with their curriculum and priorities ( Boelen *et al*., 2012). Academic contributions from institutions include developing frameworks, measurements, and methodologies that generate evidence and knowledge, seeking support from governments and other funding agencies ( Mathur *et al*., 2021). In general, HTA applies frameworks that involve mostly quantitative intervention properties as it is predominantly used in measuring clinical outcomes ( Goodman & Ahn, 1999). Such frameworks specify methods for assessing the qualities of the intervention under study, including Comparative Effectiveness Research (CER), systematic reviews, and meta-analysis on the clinical effectiveness of an intervention expressed in numbers-needed-to-treat or cost-effectiveness estimates such as incremental cost-effectiveness ratios ( Kirwin *et al*., 2022). These methodologies can be used for pricing, reimbursement, and future investments with appropriate adjustments made to take into account clinical and economic realities, as well as cultural, ethical, and philosophical considerations relevant to local policymaking for the Indian healthcare systems.

## Aim and research questions

This research aims to describe the HTA methods that are in use and the pathways for successful implementation of HTA in the WHO South-East Asia region. The following research questions will be answered through this review.

1. What are the pathways that explain the implementation of HTA in the WHO South-East Asia region?
2. What are the learnings that can be contextualized for an Indian settings?

## Methods

A traditional systematic review is specific and focuses on knowing the effectiveness of intervention(s). However, a realist review approach proposed by Pawson and colleagues (2005) helps to understand the mechanisms of complex interventions, why they worked in a particular context, and for whom ( Pawson *et al*., 2005). A variety of methods have been used to assess health interventions, and multiple factors such as access to local data, financial resources, human resources, knowledge, skills, and capacities influence the choice of a particular method ( Babigumira *et al*., 2016). Therefore, the same method may not be feasible in all the regions. A traditional systematic review alone will not be able to give us evidence of the HTA methods that were successful in various contexts. Therefore, a realist review approach will be used to understand the mechanisms of health technology interventions and the factors influencing HTA methods in different settings. This will further help policy makers to have an efficient healthcare resource allocation and strengthen evidence-based healthcare. The proposed realist review will be reported as per the Realist and MEta-narrative Evidence Syntheses: Evolving Standards [RAMESES II] guidelines ( Wong *et al*., 2017).

The steps involved in a realist review include developing a program theory, search strategy, screening and study selection, data extraction, and data synthesis. Stakeholders will be consulted at different stages of the review for their input. The individual steps are detailed below.

### Developing the program theory

**Figure 1.**
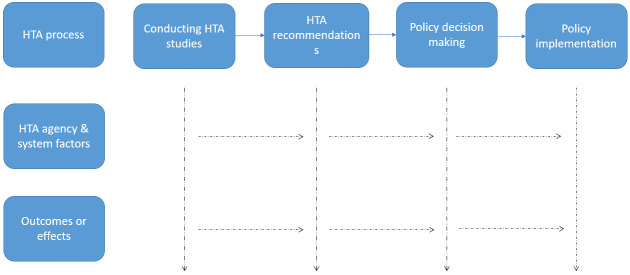
Steps involved in HTA process.

A broad search for literature will be conducted on PubMed (NCBI) and Embase (ELSEVIER). After identifying the relevant methods, we will develop an Initial Program Theory (IPT) to explain the approaches used for conducting HTA. The steps involved in HTA adapted from ( Millar *et al*., 2021) are shown in Figure 1. There is no single way to conduct HTA that will meet the needs of all decision makers, stakeholders, and societies. This is more evident in regions with decentralized healthcare systems, such as the United States and parts of Europe, where many decision-makers strive to ensure maximum use of their budgets while being restrained by multiple factors for its use and application ( Drummond *et al*., 2008). However, the review will help us understand which methods were employed, by whom, and what factors influenced the success or failure of implementing HTA for health technology interventions. Once the IPT is developed, we will undertake the literature review, and organize a consultation with stakeholders for their inputs to refine and finalize the IPT. The stakeholders will include subject experts, policy makers, researchers/academicians, and industries/companies that use HTA. All stakeholders will be allowed to decline to participate in the discussion to ensure that data collection meetings include only those who are willing to participate and provide information. Participants will be encouraged to be frank from the outset of each session, with the researcher aiming to establish a rapport in the opening moments and indicating that there are no right answers to the questions that will be asked. Where appropriate, the independent status of the researcher should also be emphasised. Participants can, therefore, contribute ideas and talk about their experiences without fear of losing credibility in the eyes of the managers of the organization. It will be made clear to participants that they have the right to withdraw from the study at any point, and they should not even be required to disclose an explanation to the investigator.

### Search strategy and information sources

We will then conduct a systematic search for literature relevant to the research question to refine the IPT. The IPT will guide the search terms. We will search for literature, which will help identify HTA methods used for health technology interventions. Examples of keywords are: ‘HTA’, ‘biomedical technology assessment’, ‘technology assessment’, ‘technological assessment’, ‘technological evaluation’, ‘economic assessment’, ‘economic evaluation’, ‘HTA model’, ‘HTA methods’, ‘HTA methods’, ‘HTA frameworks’, ‘criteria’, ‘criterion’.

The review team designed the initial search strategy with the help of a search expert to run the searches on relevant databases. An example of search strategy for Pubmed (NCBI) is given in Table 1 below. We will search multiple electronic databases such as PubMed (NCBI), EMBASE (Elsevier), Scopus (Elsevier), Web of Science (Clarivate), and ProQuest Central. The secondary source of potentially relevant material will be a search of the grey literature in the International Network of Agencies for Health Technology Assessment (INAHTA), Health Technology Assessment in India (HTAIn), Health Intervention and Technology Assessment Program (HITAP), World Health Organisation (WHO), and National Evidence-based Healthcare Collaborating Agency (NECA). We will perform hand searching of the reference lists of included studies.

**Table 1.** Search strategy

PubMed (NCBI) Date of search: 21-06-2024	No. of articles
(("structural"[Text Word] OR "structurally"[Text Word] OR "structurals"[Text Word] OR "structuration"[Text Word] OR "structurations"[Text Word] OR "structure"[Text Word] OR "structure s"[Text Word] OR "structured"[Text Word] OR "structures"[Text Word] OR "structuring"[Text Word]) OR ("conducive"[Text Word] AND "factors"[Text Word]) OR "development"[Text Word] AND ((("technology assessment, biomedical"[MeSH Terms] OR Biomedical Technology Assessment [Text Word] OR Health Technology Assessment [Text Word] OR Technology Assessment [Text Word] OR "HTA model" [Text Word] OR "Health Technology Assessment Model" [Text Word] OR "HTA values" [Text Word]) OR ("cost-benefit analysis"[MeSH Terms] OR economic evaluation [Text Word] OR Cost-Benefit Analyses [Text Word] OR Cost Benefit Analysis [Text Word] OR Cost Utility Analysis[Text Word] OR Cost-Utility Analyses [Text Word] OR Cost Benefit [Text Word] OR Marginal Analysis [Text Word] OR Cost Benefit Data [Text Word] OR Economic Evaluation [Text Word] OR Economic Evaluations [Text Word])))) AND (WHO SEARO[Text Word] OR Southeast Asia Region[Text Word] OR SEAR[Text Word] OR WHO SEAR[Text Word] OR "Bangladesh"[MeSH Terms] OR bangladesh[Text Word] OR "india"[MeSH Terms] OR India[Text Word] OR "bhutan"[MeSH Terms] OR Bhutan[Text Word] OR "indonesia"[MeSH Terms] OR Indonesia[Text Word] OR "maldives"[MeSH Terms] OR Maldives[Text Word] OR "myanmar"[MeSH Terms] OR Myanmar[Text Word] OR "Nepal"[MeSH Terms] OR Nepal[Text Word] OR "sri lanka"[MeSH Terms] OR Sri Lanka[Text Word] OR "thailand"[MeSH Terms] OR Thailand[Text Word] OR "timor-leste"[MeSH Terms] OR Timor-Leste[Text Word] OR ("republic of korea"[MeSH Terms] OR Republic of Korea[Text Word]))	381

### Screening, study selection, and appraisal

*Inclusion criteria:* We will search for English language literature published after the year 2000, since HTA in this region developed after that period. There will be no restrictions regarding the type of publication and grey literature. Studies (any study design) explaining the methods of HTA and studies explaining the implementation of HTA in the WHO SEARO region.

*Exclusion criteria:* We will exclude studies that do not provide adequate information on how the HTA was implemented; factors that influenced the implementation of HTA, and the outcomes.

Records will be managed using Rayyan software ( Ouzzani *et al*., 2016). Two reviewers will conduct the title, abstract, and full text screening independently. Studies fulfilling the inclusion criteria will be marked as ‘included’ and those not fulfilling all criteria will be marked ‘excluded’. Studies for which there is no clarity will be marked as ‘unclear’ and any disagreements will be resolved by discussion with another expert until we reach a consensus. The reasons for exclusion of the article at the full text stage will be recorded. This will ensure transparency in article selection process. The quality appraisal will be done based on the relevance and rigour of the study. We will develop a scoring criterion and cut off for including the studies and narrowing down the final subset of articles. Relevance refers to how much a study contributed to building the theory and rigour refers to how much the method used to generate data can be trusted. In addition, we will use quality assessment tools appropriate for individual study design (e.g., RoB tool for RCTs, MMAT for mixed methods, etc).

### Data extraction

The data extraction will be conducted by two authors independently. A pre-designed, pilot tested data extraction sheet will be used for data extraction by two reviewers. Any disagreements will be resolved by discussion with another reviewer until we reach a consensus. The data extraction form will include the contents of the IPT, relevant contextual factors along with article characteristics. During data extraction, if any relevant information is missing in the article, it will be marked as ‘not reported’. The data extraction format will be pilot tested on five selected articles. The data extraction sheet will include study characteristics (title, author, publication year, publication status, country, and study objectives), HTA method related details (relevance to IPT and implementation strategies), context, mechanism, outcome aspects, and quality appraisal of the study.

### Data synthesis

After the data extraction process is completed, we will develop context-mechanism-outcome configurations (CMOcs). Depending on the data available for the synthesis, we will develop visual, or narrative (thick if-then-because) or CMOcs that best synthesize the findings. The initial program theory will be reviewed and refined by comparing it with the primary evidence and exploring and analysing its relationships between contexts, mechanisms, and outcomes. The reviewers will look for recurring patterns of CMOs across the data that can support, contradict, or inform the program theory. The analysis will involve refining the IPT to identify different methods used in HTA across the globe, the mechanisms involved in implementing the HTA method, and the outcomes of the HTA of health interventions. The analysis process will be done through multiple discussions with the review team. The extracted data will be summarized into evidence tables and themes will be developed. Results will be synthesized based on ‘what works’, under ‘what circumstances’, and for ‘whom’. The results will be presented narratively. A refined program theory will be developed based on this literature review.

## Discussion

To our knowledge, there has been no comprehensive review conducted to describe the HTA methods and the pathways for implementation in the WHO South-East Asia region. The findings from the realist review will help us understand the HTA methods used in WHO South-East Asian countries and the mechanisms of the implementation of HTA methods that worked in a particular context. And why did it work? We will then contextualize the findings based on evidence feasible for Indian settings. An implementation framework will be developed that can be used by policymakers/HTA experts in India for effective implementation of HTA. The main limitation we anticipate while conducting the review is a scarcity of published data on HTA-related research. There may be undocumented or inaccessible reports on HTA websites or with relevant stakeholders. We foresee engaging with such stakeholders working in this domain to get such information. 

### Ethics and dissemination

This realist review will involve secondary data analysis of already existing data and hence ethical approval is not required. The findings from this review will be disseminated through presentations at seminars/ conferences and in peer-reviewed journals.

## Study status

The review has not yet started. We have developed a search strategy for all the databases.

## Data Availability

No data are associated with this article.
