# Supplementary material for: Health technology assessment implementation in WHO South-East Asia Region: a realist review protocol
Source: Wellcome Open Res. 2024 Jul 2;8:351. Originally published 2023 Aug 17. [Version 2] doi: 10.12688/wellcomeopenres.19619.2 (PMC11263902; doi:10.12688/wellcomeopenres.19619.2)
Supplement: Peer review history

# Reviewer report: Reviewer response for version 2

**DOI:** 10.21956/wellcomeopenres.25026.r107042

**Published:** 2024-11-20

**Authors:** Adrian Gheorghe

**Affiliations:** 1 Center for Global Development, Washington, District of Columbia, USA

Thank you for the opportunity to review this study protocol. A few points the authors may wish to consider:

- Background: authors may wish to refer explicitly to HTA progress already made in India by HTAIn and to other studies examining HTA implementation in Asia which are directly related to this study’s question e.g., Kumar et al (2022) DOI: 10.1017/S0266462322000423 At the moment the study’s premise makes it appear, by omission, as if there is no HTA progress in India, while there is quite a bit to talk about.
- Aim and research questions: I am not entirely clear what the study protocol means exactly by the terms “HTA methods” (e.g., systematic reviews, meta-analysis?), “in use” (by whom? How?), “pathways” (e.g., chronological?), “successful implementation” (does this mean application, systematic application or something else? How is success judged?) and “learnings” (what constitutes a learning?). All these terms have intuitive appeal, but they need to be defined in order to judge the appropriateness of methods.
- Methods:
  - New concepts are introduced which are not mentioned in “Aim and research questions”, namely “mechanisms”, “health technology interventions” and “factors”. Some examples are given for factors, but what do these terms mean and what is their relationship with the research questions? Moreover, it seems that the realist review will be used to “understand the mechanisms and factors” – again, how do these aims relate to the already stated “Aim and research questions”?
  - Under “Developing the program theory”, a new concept appears: “approaches [used for conducting HTA]”. Is this the same as “mechanisms”, “methods” or “pathways” above, or different? This will be important to clarify in order to understand what the IPT will be about.
  - It would be helpful to clarify the relationship between the IPT and the steps of HTA in Figure 1 – is this the expected blueprint of the IPT, or how are they linked? Moreover, the authors note that “there is no single way to conduct HTA”, which raises the risk that a single, unifying IPT may be very difficult to develop or may end up either too comprehensive or too simplistic to be useful. It may be useful for the protocol to consider how this risk will be managed.
  - The process of consulting stakeholder for the purpose of refining the IPT is unclear and, currently, cannot be reproduced based on the information provided. More details are needed on the design of the stakeholder participation exercise in order to judge its value relative to the study questions.
  - The methods focus on the first research question “what are the pathways?”, however I could see no methodological step that addresses the second questions on “learnings that can be contextualized”. How will that be done specifically?

This can be a very valuable study, not only for the SE Asia region, with a bit more clarity in aims and methods. Good luck!

Is the study design appropriate for the research question?

Partly

Is the rationale for, and objectives of, the study clearly described?

Partly

Are sufficient details of the methods provided to allow replication by others?

Partly

Are the datasets clearly presented in a useable and accessible format?

Not applicable

Reviewer Expertise:

Economic evaluation; health technology assessment.

I confirm that I have read this submission and believe that I have an appropriate level of expertise to confirm that it is of an acceptable scientific standard, however I have significant reservations, as outlined above.

## References

[1] : Assessing progression of health technology assessment implementation in Asia: a balanced scorecard for cross comparison of selected countries in Asia. *Int J Technol Assess Health Care* .2022;38(1) : 10.1017/S0266462322000423 e60 10.1017/S0266462322000423 35858879

# Reviewer report: Reviewer response for version 2

**DOI:** 10.21956/wellcomeopenres.25026.r107041

**Published:** 2024-11-18

**Authors:** Denny John

**Affiliations:** 1 Faculty of Life and Allied Health Sciences, MS Ramaiah University of Applied Sciences, Bengaluru, Karnataka, India

The search strategy does not include all search terms related to the review question. The term South East India or country names are not part of the search strategy.

 Inclusion and exclusion criteria needs to follow PICO and Healthcare Context...The current inclusion/exclusion criteria only focuses on type of literature

 Protocol should mention which of authors will contribute to which aspects such as screening, data extraction

 Critical appraisal using RAMESES is missing https://link.springer.com/article/10.1186/1741-7015-11-21 

 The authors mention that individual study design tools will be used but only mention Cochrane RoB, MMAT, however HTA reports will include cost-effectiveness analysis as well. How will this be assessed? The authors also need to use INAHTA checklist for the assessment of the HTA reports https://www.inahta.org/upload/2021/INAHTA_Checklist_anaesthesia_lens_surgery.pdf

 The protocol requires extensive language review and detailing as warranted for protocol for the subsequent version

Is the study design appropriate for the research question?

Yes

Is the rationale for, and objectives of, the study clearly described?

Yes

Are sufficient details of the methods provided to allow replication by others?

No

Are the datasets clearly presented in a useable and accessible format?

No

Reviewer Expertise:

Evidence Synthesis, Health Economics, Health Technology Assessment

I confirm that I have read this submission and believe that I have an appropriate level of expertise to confirm that it is of an acceptable scientific standard, however I have significant reservations, as outlined above.

# Reviewer report: Reviewer response for version 2

**DOI:** 10.21956/wellcomeopenres.25026.r109228

**Published:** 2024-11-18

**Authors:** Eleanor Grieve

**Affiliations:** 1 University of Glasgow, Glasgow, UK

Thank you for the opportunity to review this protocol. It sets out appropriate methods by which to help optimise the impact of HTA.  A few considerations:

**In the background section,** I feel that the description of HTA perhaps misses out on the importance of values and trade-offs. In discussing India, I would mention PM-JAY insurance as an important aspect which will come into context or mechanisms alongside HTA in addressing UHC. Also, is HTA here at the systems level i.e. country level or will you be looking at individual HTAs?

**In methods**, it would be helpful to give an example of what context and outcomes may look like. As you are searching for mechanisms, that may be less easy to show. For example, is context the type of HTA structure/governance, or country or what infrastructure/policies etc exist? Are outcomes intermediate or longer term impact.  This would help the reader understand if, for example, you are looking at mechanisms which support the uptake by practitioners of HTA recommendations into practice or perhaps at a more macro level of government buy-in.

 The theory development is an iterative process. Is its application to India to then test the theory?

 Finally, whilst this might not explicitly state realist methods, it explores mechanisms of HTA: Millar, R., Morton, A., Bufali, M.V. et al. Assessing the performance of health technology assessment (HTA) agencies: developing a multi-country, multi-stakeholder, and multi-dimensional framework to explore mechanisms of impact. Cost Eff Resour Alloc 19, 37 (2021). https://doi.org/10.1186/s12962-021-00290-8

Is the study design appropriate for the research question?

Yes

Is the rationale for, and objectives of, the study clearly described?

Partly

Are sufficient details of the methods provided to allow replication by others?

Partly

Are the datasets clearly presented in a useable and accessible format?

Not applicable

Reviewer Expertise:

Health economics / HTA

I confirm that I have read this submission and believe that I have an appropriate level of expertise to confirm that it is of an acceptable scientific standard.

# Reviewer report: Reviewer response for version 2

**DOI:** 10.21956/wellcomeopenres.25026.r88574

**Published:** 2024-07-22

**Authors:** Patricia Coelho De Soarez

**Affiliations:** 1 Departamento de Medicina Preventiva , Faculdade de Medicina da, Universidade de São Paulo, São Paulo, Brazil

I have no further comments to make.

Is the study design appropriate for the research question?

Yes

Is the rationale for, and objectives of, the study clearly described?

Yes

Are sufficient details of the methods provided to allow replication by others?

Yes

Are the datasets clearly presented in a useable and accessible format?

Not applicable

Reviewer Expertise:

Health Technology Assessment

I confirm that I have read this submission and believe that I have an appropriate level of expertise to confirm that it is of an acceptable scientific standard.

# Reviewer report: Reviewer response for version 1

**DOI:** 10.21956/wellcomeopenres.21735.r73078

**Published:** 2024-03-20

**Authors:** Cassandra Nemzoff

**Affiliations:** 1 International Decision Support Initiative,, Center for Global Development, Washington, DC, USA; 2 Global Health and Development, London School of Hygiene and Tropical Medicine, : London, UK

Thank you for the opportunity to review this interesting protocol. 

 A few small points to consider:

 - In general, your research questions are interesting, but it may be quite ambitious to answer all three of them. It may be worth considering narrowing these questions, based on the desired outcome of the research. 

 - If you are interested in capturing HTA methods which are deployed in the SE Asia region, it may be worth considering key HTA agencies' website for HTA guides and protocols. 

 - It might be helpful to consider adding search terms related to your second and third objectives, on implementation and lessons learned, to narrow what might be a quite broad literature. 

 - You may want to clarify exactly how you will use the broader HTA experiences to inform what is feasible in India to achieve your third objective, e.g will it be the authors’ judgment, or will a different approach be used to determine feasibility in India specifically?

 - A very small point, but iDSI is not a resource for searching for papers, per se. While you may find a couple of important papers published by the network on the website (I assume you might be wanting to capture "Conducive factors to the development of HTA in in Asia"), you may find more papers from the network by adding it as a search term for the published literature.

 Thanks again and good luck with your review!

Is the study design appropriate for the research question?

Yes

Is the rationale for, and objectives of, the study clearly described?

Yes

Are sufficient details of the methods provided to allow replication by others?

Yes

Are the datasets clearly presented in a useable and accessible format?

Not applicable

Reviewer Expertise:

Systematic review, economic evaluation, health technology assessment in low- and middle-income countries, 'adaptive' health technology assessment

I confirm that I have read this submission and believe that I have an appropriate level of expertise to confirm that it is of an acceptable scientific standard.

# Reviewer report: Reviewer response for version 1

**DOI:** 10.21956/wellcomeopenres.21735.r68586

**Published:** 2023-10-30

**Authors:** Patricia Coelho De Soarez

**Affiliations:** 1 Departamento de Medicina Preventiva , Faculdade de Medicina da, Universidade de São Paulo, São Paulo, Brazil

This review is relevant because it can foster HTA policy implementation in Indian settings. The authors seemed to follow the guidelines for reporting realist reviews (RAMESES II) and included the most relevant items. Some methodological steps were not presented with the necessary detail or were not mentioned in the text. I recommend the inclusion or further details of some items before its indexing. Please, find below my suggestions for improving the quality report of the manuscript.

**Methods**

- Provide details of the steps taken to enhance the trustworthiness of data collection and documentation.
- Describe how stakeholders (subject experts, policy makers, researchers/ academicians, and industries/companies who use HTA methods) will be recruited or engaged and how the sample may contribute to the development of programme theory.
- Describe in detail how data will be analysed. This section should include information on the process of analysis, how the programme theory will be further developed and refined.
- State whether the realist review required and has gained ethical approval from the relevant authorities, providing details as appropriate. If ethical approval to stakeholders consultation at different stages of the review was deemed unnecessary, explain why.

Is the study design appropriate for the research question?

Yes

Is the rationale for, and objectives of, the study clearly described?

Yes

Are sufficient details of the methods provided to allow replication by others?

Yes

Are the datasets clearly presented in a useable and accessible format?

Not applicable

Reviewer Expertise:

Health Technology Assessment

I confirm that I have read this submission and believe that I have an appropriate level of expertise to confirm that it is of an acceptable scientific standard, however I have significant reservations, as outlined above.
